# Automatic Detection of Tuberculosis Using VGG19 with Seagull-Algorithm

**DOI:** 10.3390/life12111848

**Published:** 2022-11-11

**Authors:** Ramya Mohan, Seifedine Kadry, Venkatesan Rajinikanth, Arnab Majumdar, Orawit Thinnukool

**Affiliations:** 1 Department of Computer Science and Engineering, Division of Research and Innovation, Saveetha School of Engineering, SIMATS, Chennai 602105, India; 2 Department of Applied Data Science, Noroff University College, 4612 Kristiansand, Norway; 3 Artificial Intelligence Research Center (AIRC), College of Engineering and Information Technology, Ajman University, Ajman 346, United Arab Emirates; 4 Department of Electrical and Computer Engineering, Lebanese American University, Byblos 1401, Lebanon; 5 Faculty of Engineering, Imperial College London, London SW7 2AZ, UK; 6 College of Arts, Media, and Technology, Chiang Mai University, Chiang Mai 50200, Thailand

**Keywords:** communicable disease, tuberculosis, X-ray, VGG19, Seagull-algorithm, serial concatenation, binary classification

## Abstract

Due to various reasons, the incidence rate of communicable diseases in humans is steadily rising, and timely detection and handling will reduce the disease distribution speed. Tuberculosis (TB) is a severe communicable illness caused by the bacterium Mycobacterium-Tuberculosis (M. tuberculosis), which predominantly affects the lungs and causes severe respiratory problems. Due to its significance, several clinical level detections of TB are suggested, including lung diagnosis with chest X-ray images. The proposed work aims to develop an automatic TB detection system to assist the pulmonologist in confirming the severity of the disease, decision-making, and treatment execution. The proposed system employs a pre-trained VGG19 with the following phases: (i) image pre-processing, (ii) mining of deep features, (iii) enhancing the X-ray images with chosen procedures and mining of the handcrafted features, (iv) feature optimization using Seagull-Algorithm and serial concatenation, and (v) binary classification and validation. The classification is executed with 10-fold cross-validation in this work, and the proposed work is investigated using MATLAB^®^ software. The proposed research work was executed using the concatenated deep and handcrafted features, which provided a classification accuracy of 98.6190% with the SVM-Medium Gaussian (SVM-MG) classifier.

## 1. Introduction

In recent years, the incidence rate of Infectious Disease (ID) has been gradually increasing in humans, and timely detection and treatment are essential to cure and control the spread of the disease. The ID usually affects people with a weak immune system, and this disease is commonly transmitted from one individual to another. Thus, it is regarded as a communicable disease as well. Furthermore, the ID which infects the inner body organ is harsher in comparison to the ID which infects the eternal body organ [1,2,3,4].

In hospitals, a unique diagnostic and treatment procedure is employed to cure/control the disease in internal organs infected with ID. As part of human physiology, the lung exchanges air between the outer atmosphere and other body parts, and any infection in the lung will affect this exchange. There is evidence that the lungs are commonly affected by infections such as pneumonia, tuberculosis, and COVID-19 [5,6,7]. Several diagnostic procedures exist to detect pneumonia with the help of medical images or chosen methods. Pneumonia is an infectious disease commonly affecting people with lower immunity (children and older adults). COVID-19 is also a pathogen that causes severe pneumonia in humans, and many medication procedures have been recommended to reduce the severity of COVID-19 infection in recent years [8,9,10].

As a result of TB infection, people will experience mild to severe breathing problems, and if TB is not detected, it can result in death. A common cause of tuberculosis is Mycobacterium tuberculosis (M. tuberculosis), a bacterium responsible for infecting the lungs and other soft tissues. As TB infection in the lung is a common illness, the clinical level diagnosis is performed using biomedical images of the chosen modality. A computed tomography (CT) or chest radiograph can be used to examine the lung section. X-ray images have been used in medical clinics to examine tuberculosis more frequently than CT images. Since tuberculosis is a communicable disease, and can spread quickly from one person to another, timely detection and treatment are essential [5].

As stated by the World Health Organization (WHO), tuberculosis is a serious disease and a significant cause of death worldwide. TB is considered among the top 10 deadliest diseases because the mortality rate is higher than HIV/AIDS. According to the WHO statement, 1.4 million people worldwide died of tuberculosis in 2019, while ten million cases of tuberculosis have been estimated for 2020. It is estimated that approximately 90% of the community’s adults are infected with tuberculosis (TB), with men contracting the disease at a significantly higher rate than women. Further, TB infection rates are lower in high-income countries than in low-income countries. TB will result in financial suffering, weakness, marginalization, and bias among TB-affected citizens. TB that is detected in a timely fashion is entirely curable, and >85% of those who develop TB can recover with a six-month prescription regimen [11,12,13].

Various clinical tests, including a bio-image-based examination, are used to diagnose TB at the clinical level. Using the bio-images (CT/X-ray) analysis, an experienced pulmonologist can determine the severity of the lung infection caused by tuberculosis. In addition, CT/X-ray-supported tuberculosis detection helps the pulmonologist confirm the disease’s severity, decision-making, and treatment execution processes.

There are several automated disease detection schemes proposed and employed to evaluate CT/X-rays to reduce the diagnostic burden on pulmonologists. Furthermore, modern hospitals utilize standard procedures to assist physicians during the diagnosis and treatment of diseases, including segmentation, Machine-Learning-Scheme (MLS), and Deep-Learning-Scheme (DLS) [14,15,16,17].

This research aims to develop an automated TB detection system using optimally selected Machine-Learning-Features (MLF) and Deep-Learning-Features (DLF) to examine chest X-ray images with better diagnostic accuracy. The proposed scheme consists of the following phases: (i) image pre-processing, (ii) implementing a pre-trained DLS and mining of the DLF, (iii) extracting the necessary MLF using Local-Binary-Pattern (LBP) and Discrete-Wavelet-Transform (DWT), (iv) optimization of DLF and MLF using Seagull-Algorithm (SA), (v) serial concatenation of DLF and MLF, and (vi) binary classifier implementation and validation.

In this work, 7000 test images are collected for assessment from the database supplied by Rahman et al. [18,19]. Initially, all these images are resized to 224×224×1 pixels, and the converted images are then evaluated using the pre-trained DLS existing in the literature. For the AlexNet, the chest X-ray images are resized to 227×227×1 pixels. Initially, the conventional DLF obtained from every DLS is considered to classify the chest X-ray images using the SoftMax classifier, and the performance is verified. This investigation confirms that the VGG19 helped improve classification accuracy (92.9048%). Then, this DLF is optimized using the SA and combined with the SA-optimized MLF (DLF+MLF). These concatenated features are once again considered for the classification task, and the binary classification obtained with SVM-Medium Gaussian (SVM-MG) classifier offered an accuracy of ≈98.62%. This result is then compared and validated with the earlier result by Rahman et al. [18].

The chief involvement of this research comprises.

(i) Implementation of pre-trained DLS-based TB detection from chest X-ray;
(ii) Generation of MLF using LBP and DWT;
(iii) SA-based feature optimization and serial feature concatenation to obtain DLF+MLF.

Section 2 presents earlier research, Section 3 demonstrates the methodology, and Section 4 and Section 5 demonstrate the investigational result and conclusion, respectively.

## 2. Earlier Research

Several diagnostic procedures have been proposed to reduce the diagnostic burden on hospitals to ensure early and accurate detection of tuberculosis. Several chest X-rays supported automated detection methods for tuberculosis are discussed in the literature. This research section examines DLS-based TB diagnostic procedures discussed in the literature. Table 1 summarizes the methods used to detect tuberculosis in earlier studies.

The recent research of Rahman et al. [18] confirms that the ideally employed DLS will help to support reliable TB detection from chest X-ray images. Furthermore, this work also contributed an image database with 7000 test images (3500 normal and 3500 TB class). In the proposed research, the dataset supplied by Rahman et al. [19] is considered for the experimental investigation, and the performance of the pre-trained VGG19 is improved with the help of SA-based feature optimization and serial feature concatenation among the optimized values of DLF and MLF. Further, this work also employed various recent binary classifiers discussed in the literature.

## 3. Methodology

This section presents essential information about the developed procedure to examine the chest X-ray and detect the normal/TB class images.

**Figure 1:**
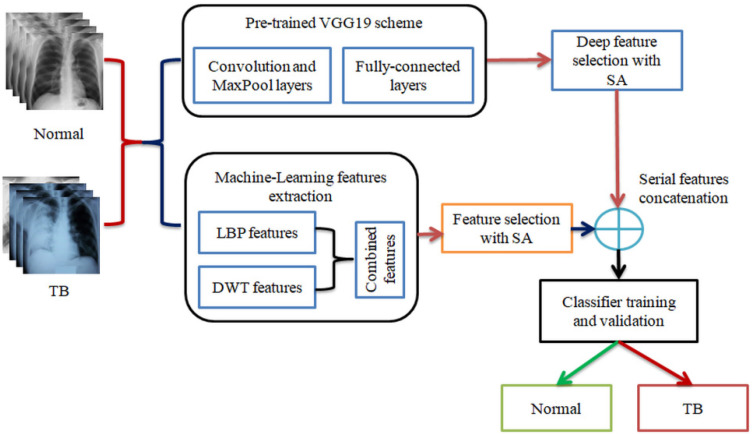
Proposed TB detection scheme from X-ray using concatenated features.

In this work, the necessary images are collected from the dataset of Rahman et al. [18] and each picture are resized to a dimension of 224×224×1 pixels. Figure 1 depicts the various stages employed in the proposed scheme. The test images are initially processed with VGG19 to obtain the necessary deep features of dimension 1×1×1024. These features are then reduced using the SA and the optimized DLF are then considered for the examination. The test images are then considered to extract the necessary MLF based on the LBP and DWT and are then combined (LBP+DWT) to obtain a single 1D feature vector and these features are then reduced using the SA to obtain the optimized MLF. The optimal values of DLF and MLF are then serially concatenated to obtain the hybrid feature vector, which is then considered to train and validate the binary classifiers considered to categorize the X-ray pictures. In this work, a 10-fold cross validation is employed to obtain better classification accuracy.

### 3.1. Image Dataset

**Figure 2:**
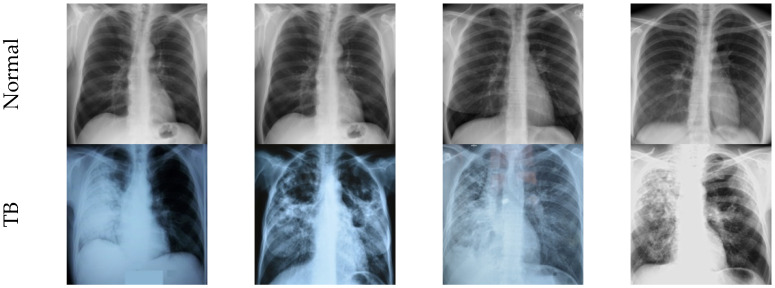
Sample X-ray pictures of healthy/TB group.

The worth of the automatic infection analysis is then confirmed using the clinical ranking or benchmark medicinal information. In this work, the chest X-ray pictures provided by Rahman et al. [19] are considered. This database consists of 7000 test images in which 3500 pictures fit in normal class and remaining 3500 is with TB traces. From these images, 70% images (2450 images) are considered as training class (in which 490 images are chosen for validation) and the remaining 30% (1050 images) are considered as testing class, as depicted in Table 2. Figure 2 presents the sample test images of the collected X-ray database.

### 3.2. Pre-Trained VGG19

DLS based clinical data evaluation is a frequently considered method and most of these approaches are used to implement automatic image categorization tasks [5,27,28,29]. In this work VGG19 is considered to examine the database and to support better TB diagnosis. Initially, the VGG19 is trained using X-ray pictures with the following tasks: (i) conventional augmentation (rotation and zoom) to boost the training images, (ii) fixing the learning rate as 1 × 10^−5^ to improve the training and validation accuracy, (iii) training with Linear-Dropout-Rate (LDR) and Adam optimization. During this task, other vital parameters are assigned as follows; total iteration = 2000, total epochs = 50, dropout rates in fully connected layer = 50%, and classification with SoftMax unit using a 10-fold cross validation.

### 3.3. Feature Extraction

This part of research outlines the extraction of DLF and MLF with chosen technique.

#### 3.3.1. Deep-Learning-Features

The proposed scheme initially aims to extract the DLF from VGG19. The final MaxPool layer section provides a feature vector of dimension 1×1×4096 and it is then passed through 3 numbers of fully connected layers with a dropout rate of 50% to obtain a reduced the DLF value to 1×1×1024. This DLF is further reduced using the SA based optimization and the reduced feature is then combined with the MLF to obtain the hybrid feature vector.

#### 3.3.2. Machine-Learning-Features

In the literature, it can be noted that the combined DLF and MLF helps to obtain a better diagnostic accuracy on various medical images and in this work, the necessary MLF is obtained using the LBP and DWT methods. Initially, the resized images are separately treated with the LBP and DWT and from these images, the necessary features are extracted.

**Figure 3:**
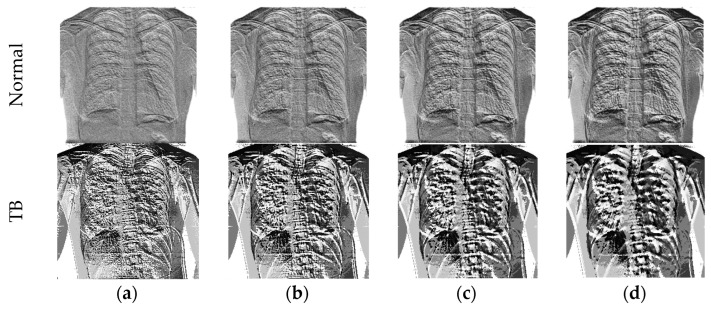
Sample test images of normal/TB class treated with LBP. (**a**) W = 1; (**b**) W = 2; (**c**) W = 3; (**d**) W = 4.

**Figure 4:**
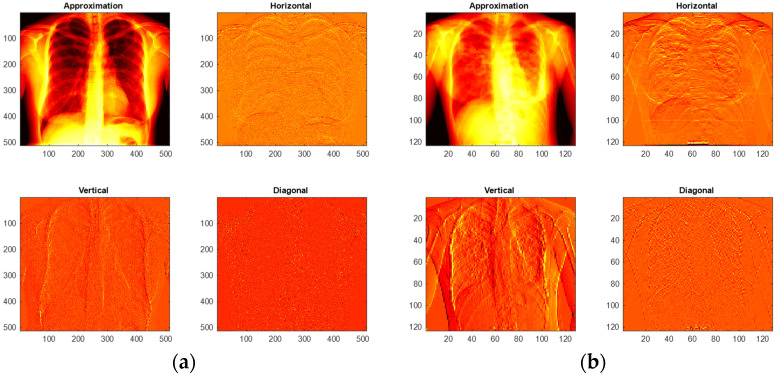
Sample test images of normal/TB class treated with DWT technique. (**a**) Normal-DWT patterns; (**b**) TB-DWT patterns.

During the LBP feature extraction task, the necessary LBP patterns are produced by assigning its weights as W=1,2,3, and 4, and from these images, essential features with a dimension of 1×1×59 are extracted from every image. The LBP technique adopted in this work is found in [30,31,32]. During the DWT feature extraction process, the test images are processed with the DWT technique discussed in [33,34,35,36,37]. This technique separates the considered test image into four equal sections, such as approximation, vertical, horizontal, and diagonal images and from every image, the necessary feature are then extracted. The extracted LBP and DWT features are then combined to obtain the original MLF, which is then reduced with the SA to obtain the optimized MLF. The necessary images of the LBP and DWT can be found in Figure 3 and Figure 4, respectively. Figure 3a–d presents LBP with various weights and Figure 4 present the results of DWT. Figure 4a,b depicts the outcome for normal and TB class images.

### 3.4. Seagull-Algorithm Based Feature Optimization

Recently, Heuristic-Algorithm (HA) based techniques are widely adopted in the literature to find the finest solution for a variety of real-world problems. The performance of any automatic data examination system depends mainly on the features considered to train and validate the classifier and to avoid the over-fitting problem, it is necessary to reduce the raw feature set. Feature selection is a recommended procedure, in which a chosen mathematical procedure (ex. Student’s *t*-test) or heuristic algorithm approach is widely adopted. The implementation of mathematical expression is widely discussed in the literature [38,39] and to automate the feature selection process, the HA based techniques are also widely considered by the researchers [40,41,42]. In the proposed work, the SA is considered to find the finest features from the raw DLF and MLF by finding the maximal value of the Cartesian Distance (CD) between the healthy and TB features.

The SA was invented in 2019 by Dhiman and Kumar [43] to find the finest solution for a chosen industrial optimization problem. This algorithm is developed by mimicking the hunting behaviors found in Seagull birds. Due to its eminence, the SA is widely adopted to solve a variety of optimization tasks [43,44,45].

**Figure 5:**
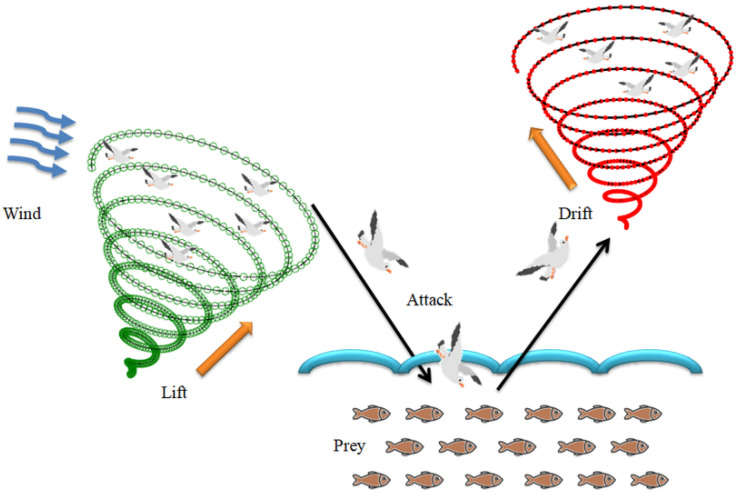
Traditional Seagull algorithm’s operation.

The basic operation in SA includes (i) migration as a group to find fish swarm (exploration) and (ii) attacking the fish (exploitation). Usually, the seagull lives as a group and always search for the food source (fish swarm) in sea surface. When it finds the food source, every seagull in the group attacks the fish swarm with its own approach. During the search, every group is led by an experienced leader and other birds will follow the leader without collision. When the group finds the fish swarm, the leader will decide the best food source and other birds follow the best source for attacking. The pictorial representation of the SA is depicted in Figure 5.

In the proposed work, traditional SA is considered for the feature selection problem and the various stages of this scheme are discussed below.

#### 3.4.1. Migration as a Group to Find Fish Swarm (Exploration)

This part of the SA helps to recognize the movement of Seagull (agent) from one location to other in search of the fish group by satisfying the conditions, such as collision prevention, association towards best neighbor and being close to the best neighbor till the food source is detected.

The collision prevention task is mathematically depicted in Equation (1):

(1) $${\vec{C}}_{s}=A\times {\vec{P}}_{s}\left(x\right)$$

where A is considered to compute the new position of agent, ${\vec{P}}_{s}\left(x\right)$ is current position, and ${\vec{C}}_{s}$ denotes the updated position.

The value of *A* is gradually varied to adjust the positions of agents and this work is mathematically denoted in Equation (2):

(2) $$A=f_{c}-\left(x\times \left(f_{c}/Iter_{max}\right)\right)$$

where $f_{c}$ = 2, $Iter_{max}$ = 3000 and $x=0,1,2,...,Iter_{max}$.

During the exploration, the movement of an agent towards the best agent can be defined in Equation (3):

(3) $${\vec{M}}_{s}=B\times \left({\vec{P}}_{bs}\left(x\right)-{\vec{P}}_{s}\left(x\right)\right)$$

where ${\vec{M}}_{s}$ is a new position of search agents is, ${\vec{P}}_{bs}\left(x\right)$ denotes fittest agent, and B is a randomization parameter and is computed in Equation (4).

(4) $$B=2\times A^{2}\times ℜ$$

where ℜ is a random value of range [0, 1].

During the exploration, every agent will closely fly with the best agent with a close distance as depicted in Equation (5):

(5) $$\mathrm{The distance}\;{\vec{D}}_{s}=\left|{\vec{C}}_{s}+{\vec{M}}_{s}\right|$$

#### 3.4.2. Attacking the Fish (Exploitation)

During this phase, the agent (Seagull) takes a spiral path to dive into the sea to catch the fish and also takes the inverted path to reach the sky from the sea as depicted in Figure 5. The operation in *x*, *y*, and *z*-plane is defined in Equations (6)–(9):

(6) $$x^{1}=r\times \cos(k)$$

(7) $$y^{1}=r\times \sin(k)$$

(8) $$z^{1}=r\times k$$

(9) $$r=u\times e^{kv}$$

where *r* is radius of the path, *k* is a random parameter [0≤k≤2π], other parameters are constants, and these values are assigned in the earlier works [43,44,45].

**Figure 6:**
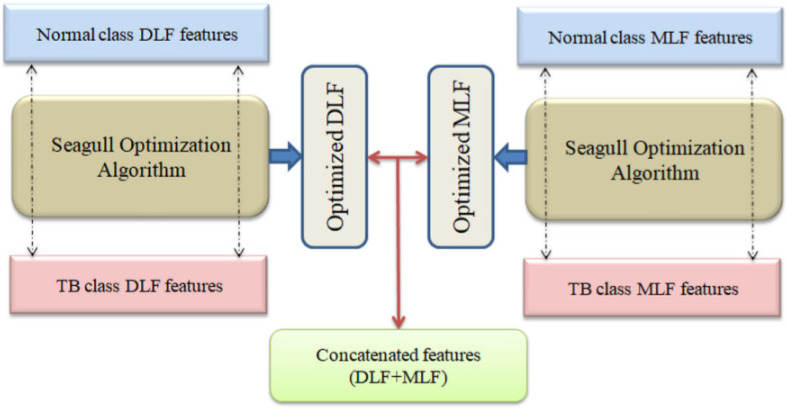
The SA based feature optimization process for DLF and MLF.

The necessary parameters of the SA are assigned as follows: number of Seagull (agent) = N = 30, search dimension (D) = 2, $Iter_{max}$ = 3000 and stopping criteria=maximization of Cartesian Distance (CD) between features or $Iter_{max}$. In this work, identification of maximized CD is chosen as the objective value and the SA helps to find the individual features of healthy/TB class whose value of CD is maximal. The SA based optimization and serial concatenation work is depicted in Figure 6. After finding the optimal features, the necessary hybrid features are obtained using the serial feature concatenation procedure.

The number of DLF and MLF considered in this work is presented in Equations (10)–(12) and in this work, the SA is then used to reduce these features to a lower value.

The number of DF and HF available for the optimization is depicted in Equations (10)–(12).

(10) $$DLF_{\mathrm{VGG}19\;\left(1\times 1\times 1024\right)}=VGG19_{\left(1,1\right)},VGG19_{\left(1,2\right)},...,VGG19_{\left(1,1024\right)}$$

(11) $$LBP_{\left(1\times 1\times 236\right)}=LBP1+LBP2+LBP3+LBP4$$

(12) $$DWT_{\left(1\times 1\times 180\right)}=DWT1+DWT2+DWT3+DWT4$$

Initially, the DLF is optimized with the SA and this process helps to reduce the feature set to a value of 1×1×539. Similar procedure is then implemented on the MLF ((1×1×236) + (1×1×180)) and this procedure helped to obtain a feature vector with dimension 1×1×107. These two features are then considered to obtain a hybrid feature as depicted in Equation (13):

(13) $${\left(DLF+MLF\right)}_{optimized}=\left(1\times 1\times 539\right)+\left(1\times 1\times 107\right)=1\times 1\times 6461\times 1\times 107$$

The feature vector shown in Eqn.13 is then used to train and validate the classifier and, in this research, well known classifiers, such as SoftMax, Naïve-Bayes, Logistic Regression, Decision-Tree (DT) variants, K Nearest Neighbors (KNN) variants, and SVM variants are considered to validate the performance of the proposed system [40,41,42].

### 3.5. Performance Validation

The merit of automatic disease discovery arrangement is to be confirmed by computing the essential performance values. In this work, the measures obtained from the confusion matrix are considered to substantiate the importance of the proposed system. These measures, includes True-Positive (TP), False-Negative (FN), True-Negative (TN), False-Positive (FP), Accuracy (ACC), Precision (PRE), Sensitivity (SEN), Specificity (SPE), and Negative-Predictive-Value (NPV). The mathematical expressions of these values are presented in Equations (14)–(18) [27,28].

(14) $$ACC=\frac{TP+TN}{TP+TN+FP+FN}$$

(15) $$PRE=\frac{TP}{TP+FP}$$

(16) $$SEN=\frac{TP}{TP+FN}$$

(17) $$SPE=\frac{TN}{TN+FP}$$

(18) $$NPV=\frac{TN}{TN+FN}$$

## 4. Results and Discussion

This section of research demonstrates the experimental result, and this work is performed by a workstation, Intel i7 2.9GHz processor with 20 GB RAM and 4GB VRAM equipped with Matlab^®^.

The resized X-ray images are initially tested using the VGG19 scheme. Initially, the pre-trained DLS is trained using the test images depicted in Table 2 and after getting the necessary accuracy, its performance is then validated with a 10-fold cross validation procedure.

**Figure 7:**
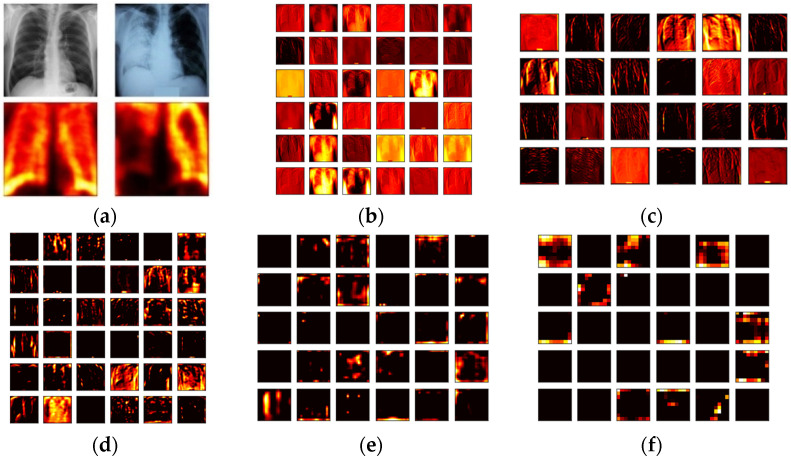
Results extracted for a sample test image with VGG19 architecture. (**a**) Heat-map; (**b**) Convolution-outcome 1; (**c**) Convolution-outcome 2; (**d**) Convolution-outcome 3; (**e**) Convolution-outcome 4; (**f**) Convolution-outcome 5.

Figure 7 depicts the outcome during the deep-feature extraction procedure with VGG19. Figure 7a presents the heat-map obtained for both the normal and TB class trial images, Figure 7b–f shows the sample outcome extracted from each convolutional layer. This procedure helps to obtain a DLF of dimension 1×1×1024. This feature vector is then considered to verify the TB detection performance of VGG19 with the SoftMax based binary classification and the attained results are shown in Table 3. This table confirms that Trial 8 helped to obtain the better classification accuracy compared to other trials. A similar procedure is then repeated with other pre-trained schemes and the attained results are presented in Table 4. The results existing in Table 4 confirm that the classification accuracy achieved with VGG19 is better compared to other methods considered for the assessment.

**Figure 8:**
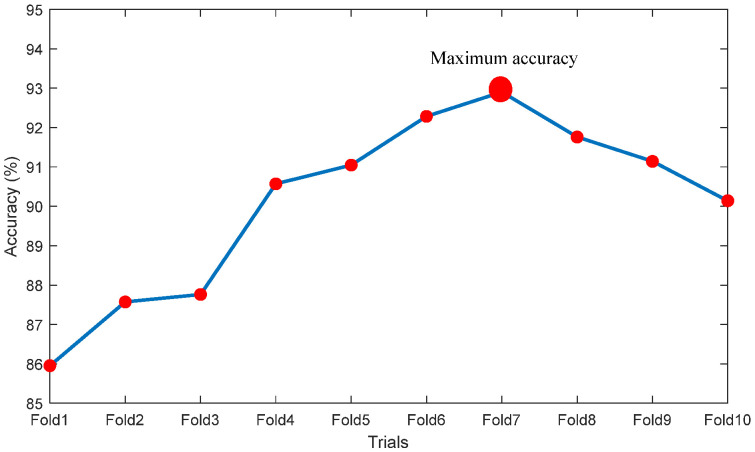
Performance evaluation of 10-fold validation and considered deep-learning systems.

**Figure 9:**
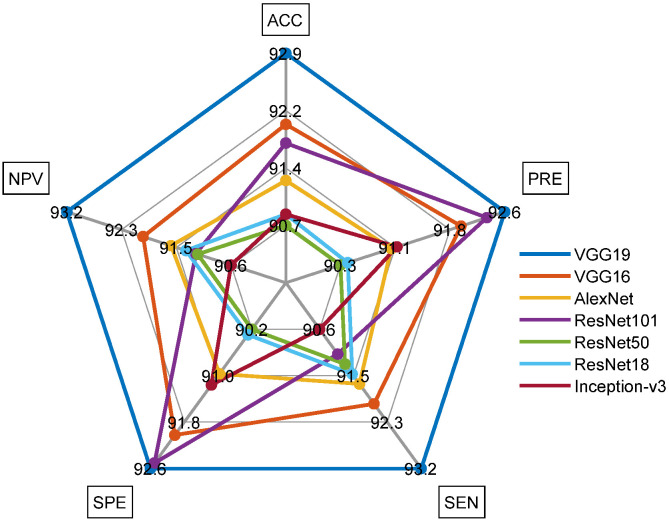
Spider-plot to demonstrate the overall performance of chosen DNN schemes.

**Figure 10:**
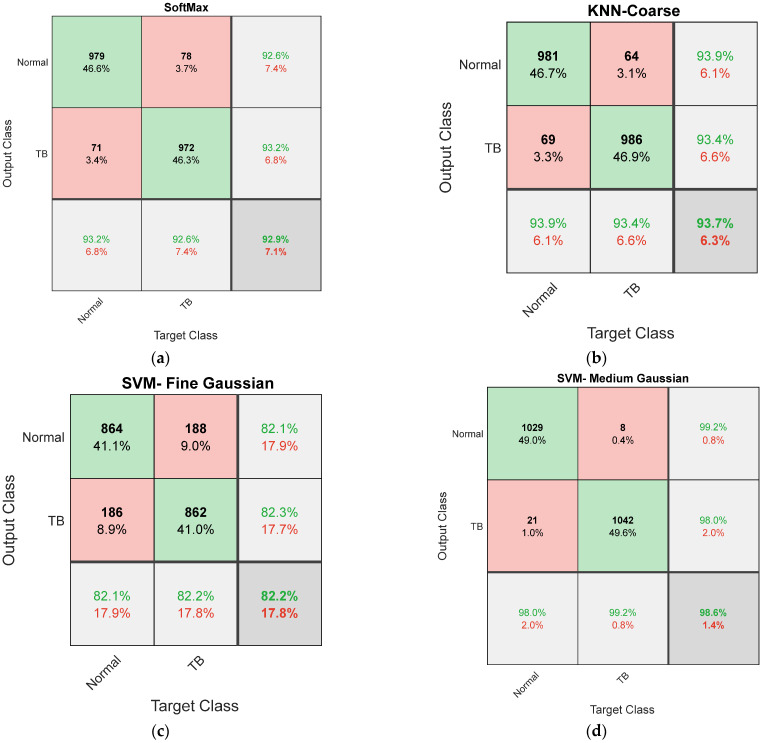
Confusion matrix achieved for VGG19 with DLF, optimal DLF, MLF, and optimized DLF+MLF.

Figure 8 graphically shows the accuracy achieved during the 10-fold cross validation and Figure 9 presents the Spider-Plot of Table 4 to verify the overall performance of the chosen schemes. The Spider-Plot is one of the graphical procedures normally considered to find the overall performance during the classification task. The plot which covers maximal area is considered superior compared to other plots. In Figure 9, it is noted that the plot area of VGG19 is more compared to other approaches, which confirms its superiority over other DLS. The obtained Confusion-Matrix (CM) for VGG19 with SoftMax classifier is depicted in Figure 10a,b presents the result by SVM- Medium Gaussian (SVM-MG) classifier.

The performance of the proposed scheme is initially verified using the optimized value of DLF and MLF, and the results are depicted in Table 5. This result confirms that the DLF-supported classification presented better accuracy (93.6667%) when considering KNN-Coarse. Similarly, the MLF-based classification achieved 82.1905% accuracy with SVM- Fine Gaussian. The confusion matrix achieved during this work is presented in Figure 10. Figure 10b,c depict the confusion matrix achieved with optimal DLF and MLF.

After confirming the performance of VGG19 with traditional features (1×1×1024), the classification of X-ray is once again repeated with the SA optimized hybrid features presented in Equation (13) (DLF+MLF). The classification achieved with the SoftMax helps to provide an accuracy of 95.09%. Further, the classification performance of VGG19 with other binary classifiers is verified using 10-fold cross validation and the attained results are presented in Table 6. This table confirms that the result obtained with SVM-Medium Gaussian (SVM-MG) classifier is better compared to other approaches and the CM attained with this classifier is depicted in Figure 10d.

**Figure 11:**
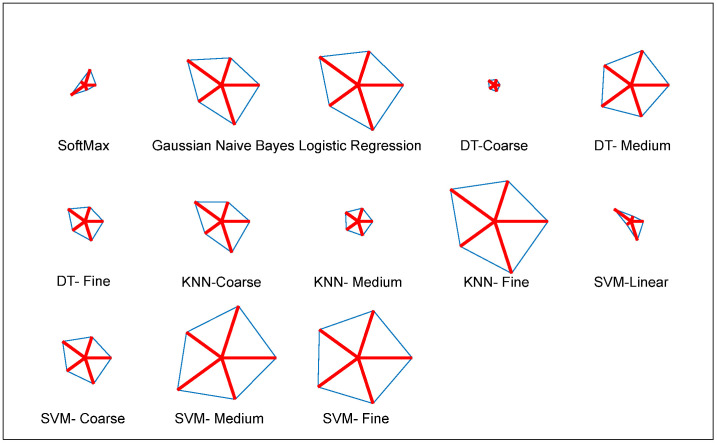
Glyph-plot constructed to demonstrate the performance for DLF+MLF.

To verify the overall performance of the results shown in Table 6, a Glyph-Plot is then constructed, and the result is demonstrated in Figure 11. This plot also confirms that the SVM-MG provides better outcome compared to other classifies adopted in this study. This result confirms that the VGG19 with concatenated feature helps to obtain a better overall result when SVM-MG classifier is employed.

**Figure 12:**
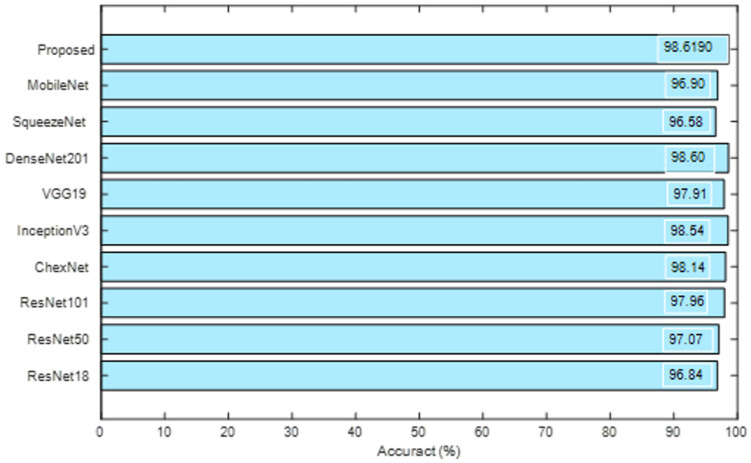
Performance comparison of proposed scheme with earlier works in the literature [18].

The merit of the proposed scheme is then confirmed with a comparative analysis with the results provided by Rahman et al. [18]. The plot depicted in Figure 12 verifies that the accuracy by the proposed system with SVM-MG classifier is very close to DenseNet201 result of [18].

This research work automatically classified X-ray images using SA-optimized hybrid image features (DLF+MLF) with a VGG19 scheme. The classification task is executed using SoftMax and other binary classifiers existing in the literature to achieve better classification accuracy. The experimental result of this work confirms that the proposed scheme works well on chest X-ray images. In the future, this scheme can be considered to evaluate the clinically collected chest X-ray images. Further, the merit of this system can be tested and confirmed using other benchmark chest X-ray pictures of different lung irregularities. The main limitation of the proposed scheme is the integration of deep and handcrafted features, and this procedure can be improved by considering the ensemble of features technique in future.

## 5. Conclusions

The literature confirms that TB is a harsh illness in human communities, which extensively infects the lungs. It is a communicable disease and, hence, premature identification, and handling is necessary to reduce the harshness. Further, appropriate detection and suggested medication will help to heal the TB completely. Due to its importance, a substantial amount of automatic TB detection is performed by the research. This research aims to propose a DLS based automatic TB detection using the concatenated DLF and MLF. This work considered VGG19 to extract the DLF and the necessary MLF are obtained using LBP and DWT approaches. After extracting these features, SA based feature optimization is employed and the optimized features are then combined to obtain a hybrid feature vector (DLF+MLF). This feature vector is then used to test and validate the performance of the binary classifiers using a 10-fold cross validation and the investigational result of this study confirmed that the binary classification with SVM-MG classifier helped to achieve an accuracy of >98% for the considered chest X-ray images. This outcome is then evaluated and against the result of other DLS found in the literature. This research confirmed the merit of the proposed DLF+MLF based TB detection from the chest X-ray images. In future, the performance of the proposed scheme can be tested and validated with other chest X-ray image dataset available in the literature.

## Figures and Tables

**Table 1:** Summary of automated TB diagnosis using chest X-ray images.

Reference	Developed Procedure
Rajaraman and Antani [20]	Implementation of modified DLS is presented in this research to inspect the Shenzhen CXR images and this scheme offered a classification accuracy of 83.7% on the considered images.
Hwa et al. [21]	Assessment of TB using the chest X-ray with ensemble DLS and Canny supported edge discovery is discussed and this investigation offered a categorization accuracy of 89.77%.
Wong et al. [22]	This research implemented a novel DLS called TB-Net to examine the TV from X-ray pictures and this work helped to obtain improved performance (accuracy = 99.86%) measure on the chosen image database.
Hooda et al. [23]	Implementation of a customized DLS with 7 convolutional (Conv) and 3 fully connected (FC) layer is discussed to recognise the TB from the chosen test pictures and this DLS offered an accuracy of 94.73%.
Rohilla et al. [24]	This work implemented pre-trained DLS (AlexNet and VGG16) to automatically detect the chest radiographs and achieved better accuracy (>81%).
Nguyen et al. [25]	Examination of X-ray pictures using the existing DLS is discussed, and its performance is compared using chest X-ray images.
Afzali et al. [26]	This scheme employed the contour-based shape descriptor practice to distinguish the TB with a better accuracy (>92%).
Rahman et al. [18]	This work employed Conv Neural Network (CNN) supported joint segmentation and classification to classify 7000 number of test images into healthy/TB class. With the proposed technique, this scheme achieved a best classification accuracy of 96.47% (without segmentation) and 98.6% (with segmentation), respectively.

**Table 2:** Chest X-ray images considered in this research.

Class	Dimension	Images		
		Total	Training	Testing
Normal	224×224×1	3500	2450	1050
TB	224×224×1	3500	2450	1050

**Table 3:** Performance values achieved with 10-fold cross validation for VGG19 with SoftMax Classifier.

Trials	TP	FN	TN	FP	ACC%	PRE%	SEN%	SPE%	NPV%
Fold1	892	158	913	137	85.9524	86.6861	84.9524	86.9524	85.2474
Fold2	907	143	932	118	87.5714	88.4878	86.3810	88.7619	86.6977
Fold3	904	146	939	111	87.7619	89.0640	86.0952	89.4286	86.5438
Fold4	958	92	994	106	90.5714	90.0376	91.2381	89.9048	91.1197
Fold5	964	86	948	102	91.0476	90.4315	91.8095	90.2857	91.6828
Fold6	973	77	965	85	92.2857	91.9660	92.6667	91.9048	92.6104
Fold7	979	71	972	78	92.9048	92.6206	93.2381	92.5714	93.1927
Fold8	961	89	966	84	91.7619	91.9617	91.5238	92.0000	91.5640
Fold9	954	96	960	90	91.1429	91.3793	90.8571	91.4286	90.9091
Fold10	942	108	951	99	90.1429	90.4899	89.7143	90.5714	89.8017

**Table 4:** TB detection performance of the chosen DLS with SoftMax unit.

Scheme	TP	FN	TN	FP	ACC%	PRE%	SEN%	SPE%	NPV%
VGG19	***979***	***71***	***972***	***78***	***92.9048***	***92.6206***	***93.2381***	***92.5714***	***93.1927***
VGG16	966	84	966	84	92.0000	92.0000	92.0000	92.0000	92.0000
AlexNet	962	88	955	95	91.2857	91.0123	91.6190	90.9524	91.5628
ResNet101	956	94	971	79	91.7619	92.3671	91.0476	92.4762	91.1737
ResNet50	958	92	947	103	90.7143	90.2922	91.2381	90.1905	91.1453
ResNet18	960	90	948	102	90.8571	90.3955	91.4286	90.2857	91.3295
Inception-v3	951	99	957	93	90.8571	91.0920	90.5714	91.1429	90.6250

**Table 5:** Performance of proposed framework with DLF and HCF.

Feature	Classifier	TP	FN	TN	FP	ACC%	PRE%	SEN%	SPE%	NPV%
DLF	SoftMax	973	77	978	72	92.9048	93.1100	92.6667	93.1429	92.7014
	Gaussian Naive Bayes	975	75	980	70	93.0952	93.3014	92.8571	93.3333	92.8910
	Logistic Regression	976	74	976	74	92.9524	92.9524	92.9524	92.9524	92.9524
	DT-Coarse	982	68	979	71	93.3810	93.2574	93.5238	93.2381	93.5053
	DT-Medium	981	69	983	67	93.5238	93.6069	93.4286	93.6190	93.4411
	DT-Fine	978	72	980	70	93.2381	93.3206	93.1429	93.3333	93.1559
	KNN-Coarse	981	69	986	64	93.6667	93.8756	93.4286	93.9048	93.4597
	KNN-Medium	974	76	979	71	93.0000	93.2057	92.7619	93.2381	92.7962
	KNN-Fine	977	73	982	68	93.2857	93.4928	93.0476	93.5238	93.0806
	SVM-Linear	982	68	977	73	93.2857	93.0806	93.5238	93.0476	93.4928
	SVM-Coarse Gaussian	980	70	983	67	93.4762	93.6008	93.3333	93.6190	93.3523
	SVM-Medium Gaussian	979	71	977	73	93.1429	93.0608	93.2381	93.0476	93.2252
	SVM-Fine Gaussian	982	68	981	69	93.4762	93.4348	93.5238	93.4286	93.5176
MLF	SoftMax	841	209	864	186	81.1905	81.8890	80.0952	82.2857	80.5219
	Gaussian Naive Bayes	853	197	848	202	81.0000	80.8531	81.2381	80.7619	81.1483
	Logistic Regression	856	194	851	199	81.2857	81.1374	81.5238	81.0476	81.4354
	DT-Coarse	849	201	862	188	81.4762	81.8708	80.8571	82.0952	81.0913
	DT-Medium	853	197	859	191	81.5238	81.7050	81.2381	81.8095	81.3447
	DT-Fine	863	187	861	189	82.0952	82.0342	82.1905	82.0000	82.1565
	KNN-Coarse	865	185	852	198	81.7619	81.3735	82.3810	81.1429	82.1601
	KNN-Medium	862	188	861	189	82.0476	82.0171	82.0952	82.0000	82.0782
	KNN-Fine	857	193	864	186	81.9524	82.1668	81.6190	82.2857	81.7408
	SVM-Linear	853	197	863	187	81.7143	82.0192	81.2381	82.1905	81.4151
	SVM-Coarse Gaussian	858	192	862	188	81.9048	82.0268	81.7143	82.0952	81.7837
	SVM-Medium Gaussian	861	189	864	186	82.1429	82.2350	82.0000	82.2857	82.0513
	SVM-Fine Gaussian	864	186	862	188	82.1905	82.1293	82.2857	82.0952	82.2519

**Table 6:** Experimental outcome achieved with various binary classifiers.

Classifier	TP	FN	TN	FP	ACC%	PRE%	SEN%	SPE%	NPV%
SoftMax	993	57	1004	46	95.0952	95.5727	94.5714	95.6190	94.6277
Gaussian Naive Bayes	1026	24	1015	35	97.1905	96.7012	97.7143	96.6667	97.6901
Logistic Regression	1031	19	1022	28	97.7619	97.3560	98.1905	97.3333	98.1748
DT-Coarse	995	55	993	57	94.6667	94.5817	94.7619	94.5714	94.7519
DT-Medium	1018	32	1023	27	97.1905	97.4163	96.9524	97.4286	96.9668
DT-Fine	1007	43	1002	48	95.6667	95.4502	95.9048	95.4286	95.8852
KNN-Coarse	1018	32	1007	43	96.4286	95.9472	96.9524	95.9048	96.9201
KNN-Medium	1002	48	1001	49	95.3810	95.3378	95.4286	95.3333	95.4242
KNN-Fine	1037	13	1029	21	98.3810	98.0151	98.7619	98.0000	98.7524
SVM-Linear	1007	43	992	58	95.1905	94.5540	95.9048	94.4762	95.8454
SVM-Coarse Gaussian	1013	37	1009	41	96.2857	96.1101	96.4762	96.0952	96.4627
SVM-Medium Gaussian	***1029***	***21***	***1042***	***8***	***98.6190***	***99.2285***	***98.0000***	***99.2381***	***98.0245***
SVM-Fine Gaussian	1031	19	1036	14	98.4286	98.6603	98.1905	98.6667	98.1991

## Data Availability

The images considered in this work can be accessed from https://ieee-dataport.org/documents/tuberculosis-tb-chest-x-ray-database. Working video and MATLAB code: https://drive.google.com/file/d/1JfxprZHldYfgeft6iCGP1YxH4cF5-BKR/view?usp=share_link.
